# Normative account of Islamic bioethics in end-of-life care

**DOI:** 10.1080/11287462.2022.2118977

**Published:** 2022-12-06

**Authors:** Ezieddin Elmahjub

**Affiliations:** Associate Professor of Law, Qatar University, Doha, Qatar

**Keywords:** Islamic bioethics, End-of-life-care, bioethics, normative ethics

## Abstract

This article addresses the bioethical challenges raised by end-of-life care (EoLC) from the perspective of Islamic normativity. Rejecting positivist positions, it argues for the use of a flexible approach midway between a deontological conception of human life as having a sacred value that cannot be bargained over, as represented by the teachings of Abū Ḥāmid al-Ghazālī's, and one that introduces considerations of pain (alam) and pleasure (ladhdah) into ethical evaluations, as expounded by the jurist Fakhr al-Dīn al-Rāzī. Under this approach, described as “Islamic evaluator relativity,” moral agents formulate a normative position tailored to their beliefs and the circumstances of the case, in which the right course of action is expressed as a value judgement (amr ijtihādi) and the evaluator (mujtahid) is rewarded regardless of the choices they make. Keywords: Islamic bioethics, End-of-life-care, bioethics, normative ethics.

## Introduction

Advancements in medical technology have greatly reshaped approaches to death, the dying process, and the provision of end-of-life care (EoLC). Even when a medical diagnosis or prognosis has determined that a patient is terminally ill due to an incurable disease, death may be kept at bay by various means, including medication, mechanical ventilation, and artificial nutrition and hydration. A person receiving EoLC, even when in a critical condition, continues to have a moral existence, nonetheless. In such situations, ethicists seek to establish what is good and right by weighing the ethical value of human life against the pain and suffering of patients, their quality of life, financial and emotional burdens to their families, and costs to healthcare resources. Healthcare providers, patients, and their families face challenging moral dilemmas in relation to EoLC. Human life is at stake, and stakeholders often find themselves having to make moral choices: Is an act or an omission that allows death to occur the right course to follow? Is it morally preferable to prolong life regardless of the consequences for the patient and the healthcare system?

Dominant thinking in secular bioethical discourse posits that patients are autonomous moral agents with total control over their destiny, and thus, as far as is possible, they should have the final say on sanctioning or even performing acts or omissions to end their lives. Where this is not possible, healthcare providers and surrogate decision-makers may refer to normative principles when engaging in consequentialist calculations of harm and benefit to determine the right course of action. Suppose medical advice concludes a patient is terminally ill and no treatment is possible. Physicians may be of the opinion that more harm than good would be done by keeping the patient alive through medication, further procedures, or medical devices. The right course of action would then be that which allows death to happen.

In Islamic doctrine on moral obligations, the ethical challenges posed by EoLC remain largely unaddressed – at least, not directly. Guidance can nonetheless be derived indirectly from the fact that the doctrine portrays human life as having supreme ethical value. Muslim jurists are unanimous in thinking that the Islamic system of ethics is characterized by a vision in which the preservation of human life becomes in itself an end or objective (*maqsad*) that should inform and orient moral reasoning. In other words, they assert the normative character of the duty to preserve human life. In principle, when Muslims are faced with the need to reach an Islamic moral judgement (*ḥukm al-sharʿi*) on questions of life and death, they should determine that the right thing to do is to preserve human life and that it is morally reprehensible to engage in acts or omissions that would result in the termination of life. However, this strict obligation to protect life may be a cause of great moral distress for the physicians, patients, and surrogate decision-makers involved in EoLC. For instance, literally understood, the duty to preserve life means that doctors have an imperative obligation to heal. However, if the doctors agree that a patient is terminally ill and that there is very good reason to withhold or withdraw all medical interventions and let the patient die, does this prognostic supersede the duty to heal? In other words, does the overarching obligation to preserve life translate into an extended commitment to preserve life at all cost and in all circumstances?

This article starts with the proposition that, in the Islamic worldview, EoLC does not deprive human life of its moral existence, with the consequence that its sanctity must be safeguarded. When discussing EoLC in an Islamic context, it is an essential preliminary, therefore, to delineate the scope of the duty to preserve life, before physicians, patients, and surrogate decision-makers can engage in harm and benefit analysis to decide on the right course of action to take. Existing literature draws on premodern Islamic legal theory and paradigm cases in proposing practical solutions for dealing with terminal illness. However, it offers little guidance on what to make of the paramount duty to respect the sanctity of human life.

The research underlying the article began with a scoping review (Daudt et al., 2013) of the literature on EoLC in Islamic sources relevant to EoLC. The review took in sources in both Arabic and English, with particular attention being given to modern fatwas issued by prominent Islamic institutions, including the Council of Islamic Jurisprudence, al-Azhar, the International Islamic Fiqh Academy, and the European Council for Fatwa and Research. An analysis of these sources revealed that the predominant approach adopted in Islamic bioethical discourse on EoLC is to start from a position of legal positivism – that is to say, reasoning that relies on the existence of a rule already established as part of premodern Islamic jurisprudence. For instance, jurists justify the permissibility of acts or omissions leading to the death of terminally ill patients by referring to premodern opinions on the moral status of medical treatment (*tadāwī*), according to which medical treatment is not obligatory in all cases; in particular, it is not mandatory for patients who are terminally ill. In other words, it is assumed that from premodern jurisprudence a rule has been inferred which affirms the optional character of the *tadāwī*, and that rule is then applied to a wide range of bioethical challenges in EoLC. Yet, in the context of current bioethical deliberations, very little is said about the rule's merits vis-à-vis broader ethical and normative precepts in Islamic systems of ethics, including the moral value of human life. In this sense, Islamic positivism stands in the tradition of Austinian legal positivism, which separates law and morality. As Austin wrote, “the existence of law is one thing; its merit and demerit another. Whether it be or be not is one enquiry; whether it be or be not conformable to an assumed standard, is a different enquiry.” (1832/1995, p. 157).

I take issue with the reliance on positivist reasoning to formulate Islamic responses to bioethical challenges in relation to EoLC. When analyzing and addressing the ethical challenges posed by EoLC. Islamic bioethical discourse should rather consider the sources of Islamic moral obligation from an ethical perspective. The methodology I am proposing follows the structure of normative theories (Dreier, 1993). This article shows that it is possible to construct broad normative Islamic principles for the purpose of assessing, justifying, and governing ethical decisions related to EoLC. This possibility has been overlooked in existing Islamic bioethical reasoning, which uses paradigm cases to determine the permissibility of a given act or omission related to EoLC. From a normative perspective, there is no good reason why ethical interrogations concerning EoLC should necessarily be articulated in positivist language of the kind: Is X permitted or prohibited, and under what conditions? Preformed ethical responses are to be avoided. Instead, I advocate relying on Islamic legal theory (i.e. *maqāṣid al-Sharīʿah*) to construct a flexible normative methodology for dealing with EoLC ethics.

The crux of my argument is that Islamic moral norms cannot be derived exclusively from positivist reasoning based on an inflexible reading of textual sources and the application of paradigm cases. Since the 10th century CE, jurists have proposed a normative reading of the textual sources through *maqāṣid al-Sharīʿah* (normative objectives of textual sources deemed to be objectives of Islamic law). I follow leading scholars of *maqāṣid al-Sharīʿah* in showing that Islamic sources of moral obligation offer two broad normative approaches to ethical questions bearing on the value of human life. The first is rooted in a fundamental deontological conception of human life as a sacred value that cannot be bargained over in consequentialist pain/pleasure or cost/benefit calculations. This general deontic vision can be located in Abū Ḥāmid al-Ghazālī's perception of the ethical value of life. The second approach, while still stressing the importance of human life, uses a consequentialist method to assess its value. In doing so, it introduces considerations of pain (*alam*) and pleasure (*ladhdah*) into ethical evaluations. In this approach, a jurist reasons in terms of what will maximize good (pleasure) and minimize evil (pain). The jurist Fakhr al-Dīn al-Rāzī, for example, was an early exponent of this normative approach. I propose an approach to EoLC ethics that lies between these two competing positions. When facing ethical dilemmas in EoLC, moral agents could strike a balance between these two approaches by formulating a normative position tailored to their beliefs and to the circumstances of the case. Here, the right thing to do will be framed as a value judgement (*amr ijtihādi*) and, as a famous Islamic maxim has it, the evaluator (*mujtahid*) will be rewarded regardless of the choices they make. To describe this midway approach, I will use the expression “Islamic evaluator relativity.”

In comparative ethics, the notion of evaluator relativity has started to gain popularity as a means of determining the moral status of acts and omissions. The praiseworthiness or blameworthiness of certain value judgements will sometimes depend on the identity of the evaluator. For instance, if the morally required choice for some is to save a greater number of lives, we cannot blame a father for choosing to save his son even if this leads to the death of more persons. Evaluator relativity recognizes the father as a moral agent, acknowledges his wish that his child be saved, and affirms that this wish has superior ethical value over the abstract aim of saving lives unrelated to a specific moral agent (Sen, 1983).

## End-of-life medical conditions in an ethical context

EoLC is full of agonizing moral dilemmas. Modern medical technology and biochemistry developments have made it possible to relieve pain and sustain cardiac and respiratory activities and organ functions. Patients who suffer from life-limiting injury or sickness can be kept alive by providing them with the ability to breathe, digest food, and communicate with others. While technology may offer a means of sustaining patients’ lives, it does not necessarily cure terminal illnesses. Treatment for patients in EoLC is typically classified as futile. Futility generally means that recovery is improbable and that the quality of life and benefit to the patient is unacceptably low (O’Connor et al., 2011; White et al., 2016). Depending on the nature of illness or injury, the patient in EoLC may be conscious and able to express their feelings and desires about the need to continue with the treatment. For instance, patients who have quadriplegia or are suffering from end-stage metastatic cancer may still be able to communicate with their families and healthcare professionals. Other patients may be unconscious and have lost their communication ability because of brain injury or advanced dementia.

Patients in EoLC are generally admitted to palliative care, which, according to the World Health Organization, is not intended to hasten or postpone death but to improve the quality of life by alleviating pain and other distressing symptoms (World Health Organization, n.d.). Life in the EoLC environment, even with the most advanced palliative technologies, can still impose significant hardships and deplete health resources and family funds. On top of that, the prospects of returning to a healthy life can be very slim. Bioethical discourse on EoLC has traditionally sought to address the following questions: What should we make of the moral obligation to preserve life in EoLC? Does it include an ancillary duty to prolong the life of terminally ill patients? Is it ethical for the patient, healthcare providers, and families to ask or engage in acts or omissions that eventually lead to death?

The different scenarios posed by EoLC led ethicists to investigate the moral status and ethical justifications for euthanasia, physician-assisted suicide, and withholding or withdrawing medical treatment, among other issues. Ethicists typically start from the normative framework provided by Beauchamp and Childress. They suggest that, in general, bioethical interrogations are best addressed through what they call “common morality,” which they describe as “a range of norms that all morally serious persons share” (2008, p. 3). These norms manifest themselves in four normative principles. The first is the principle of autonomy, which requires decision-makers to respect the individual's right to self-governance. The second is nonmaleficence, a normative principle that requires evaluators to minimize harm. The third is beneficence, a normative principle that requires evaluators to maximize benefits. And the fourth is justice, a set of norms designed to ensure fair distribution of benefits, risks, and costs (2008, p. 12).

In EoLC, if the patient is conscious and is (was) able to communicate their desire through an immediate request or directives in advance, they can decide to withhold or withdraw medical treatment. Moral judgements of this sort are usually justified by reference to the principle of autonomy. A conscious patient with legal capacity has the power to decide their fate, and healthcare professionals cannot impose medical treatment that the patient rejects. In the judicial context, courts in the West routinely emphasize the normative importance of the principle of autonomy, with some courts declaring that the principle of autonomy prevails over the sanctity of life (e.g. *Brightwater Care Group v. Rossiter* [2009] WASC 229 (Austl.)). Furthermore, those who stretch the principle of autonomy to its fullest extent would argue that there are legitimate moral grounds for patients requesting euthanasia or medical assistance to commit suicide if their quality of life is no longer acceptable to them (De Haan, 2002, p. 154; Gorsuch, 2000, p. 657). As for unconscious or mentally impaired patients, the principles of nonmaleficence, beneficence, and justice have traditionally been used to justify decisions to continue or cease medical treatment. These principles provide normative guidance that enables decision-makers to engage in a utilitarian analysis weighing harm and benefit for the patient in terms of the prospect of recovery, levels of pain, and quality of life. They also enable decision-makers to consider the efficient allocation of healthcare resources to ensure they are used in the most productive manner. For instance, if the patient's desires are unknown, the family can decide on medical treatment even if this leads to death. Moreover, physicians can unilaterally withdraw or withhold medical treatment that they consider futile to the patient and wasteful of medical resources.

## Islamic responses

The developing field of Islamic bioethical literature as it stands today does not offer a general normative theory for analyzing the complex practical scenarios of EoLC. This lack of general normative standards becomes particularly noticeable if we compare the process of reasoning over EoLC in Islamic discourse with comparative bioethical discourse. As mentioned above, comparative bioethical literature followed Beauchamp and Childress's lead in adopting an overarching theoretical justification for bioethical decision-making based on the notion of common morality and the four principles of biomedical ethics. The process of moral reasoning in comparative bioethical moral reasoning starts from a general theory of ethical value. It then proposes normative principles that ultimately guide decision-making at the level of individual cases.

As far as Islamic bioethical reasoning is concerned, this broad form of normative analysis has yet to be incorporated in Islamic discourse on the ethical challenges of EoLC. The current process of reasoning is primarily positivist, not normative. Responses to ethical challenges in EoLC are fashioned using classic paradigm cases. Moral judgements are made on the basis of a deductivist approach rather than a normative vision that would allow flexibility in responding to the complex and evolving ethical challenges raised by EoLC. That said, a strand can be discerned in Islamic literature on bioethics which seeks out conceptual similarities between the four principles and established Islamic norms and maxims. For instance, authors such as Mustafa (2014) and Serour (1994) have suggested that Islamic juridical rules on averting harm and causing good show that Islamic jurisprudence recognizes a normative authority equivalent to the considerations underpinning Beauchamp and Childress's four principles. However, these authors do not seem to view Islamic jurisprudence as an ethical discourse in which human life is seen as a central ethical value engendering complex and conflicting normative positions.

Before critically assessing the positivist thesis of Islamic bioethics, it is important to point out that the principle of autonomy has very little normative force in dictating Islamic ethical responses to EoLC issues (cf. Sachedina, 2006). This is perhaps the most fundamental difference between Islamic and Western philosophy on bioethics. In general, the notion of autonomy is not central to the Islamic ethics system. While human life has an intrinsic ethical value, it does not seem to extend to an ancillary recognition of an unlimited right to self-governance. A human being is not empowered to make decisions to end their life. In Islamic theology, God is the ultimate owner of life, and the human is a mere trustee. In principle, subjective assessment of one's medical condition, quality of life, levels of pain, and financial costs cannot justify decisions that lead to the ending of one's life. Accordingly, individual Islamic scholars and institutions unanimously agree on a categorical prohibition on acts and omissions leading to or precipitating death or assisting the patient to commit suicide. This strict normative vision persists even when the patient is terminally ill, as affirmed by the Islamic Organization of Medical Sciences (1981) and in the resolutions adopted at the 11th session of the European Council for Fatwa and Research and Research (ECFR), Stockholm, Sweden, July 1–7, 2003, and the 22nd session of the Islamic Fiqh Academy (IFA), Kuwait City, May 10–13, 2015. Individual human perception or moral intuition cannot dictate morally required choices as far as human life is concerned. Existing Islamic biomedical discourse would only accept an objective medical assessment of futility as an empirical authority for the right action.

Medical futility is taken to be inevitable in cases of brain death. Since 1986, when the Council of Islamic Jurisprudence declared in its resolution on resuscitation apparatus that doctors may cease all medical intervention without fear of religious responsibility in the event of total cessation of all cerebral functions and where such cessation is irreversible, there would seem to be agreement that the patient is already dead and that there is no life to preserve through medical treatment. However, moral uncertainty frequently arises, with greater intensity, when evaluating the ethical status of withdrawing and withholding medical treatment for patients who are deemed terminally ill. A “terminally ill patient” is an open-ended concept that covers a wide range of medical conditions. In any given terminal illness situation, stakeholders are dealing with a human life, the preservation of which is a moral duty. At the same time, pressing ethical questions arise about the right course of action to take in the light of calculations of harm/benefit to the patient, their family, and the healthcare system. Here, as I show below, Islamic bioethical discourse could benefit from a philosophically grounded ethical theory on the basis of which to evaluate and justify the right conduct.

There is a line of thought which considers it impermissible to withhold or withdraw medical treatment from moribund patients, viewing this act or omission as a violation of the categorical moral duty to preserve human life (Ghaly et al., 2018, p. 45, 46; citing Jabbūrī, 2015). Likewise, when asked about withdrawing medical treatment from terminally ill patients at the 23rd Intentional Medical Conference of ʿAin Shams University, February 21–24, 2000, the Grand Imam of al-Azhar, Sheikh Muhammad Tantawy, responded that it was prohibited as a form of euthanasia and that neither terminal illness nor the futility of a case could justify active termination of medical treatment (al-Jufaiyyrī, 2008). The idea that the patient is dying due to the underlying medical condition seems to be ignored; instead, there is a focalization on the causal link between stopping medical treatment and the patient's death. The argument – not unlike that in comparative bioethical discourse (e.g. McGee, 2011, p. 469; Rachels, 2001, p. 949; Winkler, 1995) – is that the patient stays alive as long as they are provided with life-saving means and thus their death results from withdrawing those means. Such accounts give the moral duty to preserve life normative priority over utilitarian considerations based on harm and benefit. Accordingly, the act of withdrawing or withholding medical treatment that leads to death is considered as blameworthy as intentional killing. According to Ghaly et al. (2018, p. 29, citing Jabbūrī), this ethical position was considered applicable even to brain-dead patients in the eyes of those scholars who stressed the sanctity of human life and believed that its preservation was an obligation imposed by God.

However, the dominant position in Islamic bioethical responses, from both individual scholars and institutions, considers it permissible to withdraw or withhold medical treatment for “futile cases.” According to Yusuf al-Qaraḍāwī (2013), if specialist doctors decide that a person is terminally ill and that life-saving medical treatment is not possible, the doctors have no duty, under Islamic law, to continue with medical treatment. Al-Qaraḍāwī's opinions came in a short fatwa and included a statement affirming their general applicability to all cases that doctors deem medically futile. Similar positions were taken by al-Bār (2015) and by Permanent Committee for Research and Fatwa in Saudi Arabia, whose 1989 fatwa 12086 stated that if three competent specialist physicians are of the opinion that resuscitative measures would be useless and inappropriate for a certain patient, then there is no need for those measures to be carried out. Some international institutions, including the Islamic Fiqh Academy at its 22nd session in 2015 and the European Council for Fatwa and Research at its 11th session in 2003, have similarly accepted that Muslims have no religious duty to treat terminally ill patients and that withdrawing or withholding medical treatment is permissible, subject to confirmation of the futility of the treatment by three physicians.

Typically, when justifying the permissibility of withholding or withdrawing medical treatment, jurists consult premodern juridical discourse on *tadāwī* (the moral status of seeking medical treatment). References are normally made to al-Ghazālī's opinion in *Iḥyāʾ ʿulūm al-dīn*. Al-Ghazālī holds that: “it is permissible to forgo medical treatment if a person is moribund” (*Iḥyāʾ ʿulūm al-dīn*, n.d., p. 287). However, the bulk of modern Islamic bioethical literature cites Ibn Taymiyyah's discussion of prevailing thinking on *tadāwī* in classic Islamic legal theory. According to Ibn Taymiyyah, seeking medical treatment is not always obligatory. *Tadāwī* could only be obligatory to Muslims when there is some certainty (*qaṭ*ʿ) or high probability (*ẓann rājiḥ*) that it is life-saving. However, *tadāwī*'s ethical status shifts from obligatory to permissible when it is futile (Ibn Taymiyyah, *Majmūʿ Fatāwā al-Kubra*, n.d., p. 260). In such cases, physicians, patients, and families can engage in acts and omissions to terminate medical treatment, even if this leads to certain death.

It should be noted that jurists justify their position on stopping medical treatment by relying on rudimentary deductive reasoning from paradigm cases in a unidirectional way. They cite and take for granted the rule that *tadāwī* is not obligatory. Then, they apply this rule at the case level to justify medical decisions that affect treatment in EoLC. The ethical justification for the rule on *tadāwī* finds its primary source (though not the only one) in the exemplary behaviors of the Prophet's companions. Jurists typically argue that Abū Bakr or Muʾādh ibn Jabal rejected medical treatment for moribunds (al-Bār, *Ahkâm al-Tadāwī*, 1995, pp. 39–41). Therefore, Muslims can make value judgements about their EoLC decisions, including decisions that lead to terminating life-sustaining measures.

However, this unidirectional deductivism fails to provide a general normative framework for justifying ethical judgements in EoLC. First, Islamic legal theory does not normally place the exemplary behavior of the companions as an unquestionable source for moral actions. Jurists of *uṣūl* have usually debated the organizing authority of this source. This is particularly true when dealing with complex ethical situations that lack clear scriptural guidance, as is the case for EoLC (al-Bagha, *Athar al-Adillah*, n.d., p. 339). Accordingly, it would be contrary to the general framework of Islamic legal theory to suggest that positivist reasoning from paradigm cases on *tadāwī* is sufficient to address EoLC challenges. Second, if we follow the normative reading for the value of human life that will be presented below, it would be challenging to accept the proposition that *tadāwī* should be viewed as optional. This form of classic positivism will have us accept isolated practices of companions as authority for the right action without further investigation. There is room to assess the positivist rule on *tadāwī* through a general normative vision that I aim to construct in this article. For instance, how can we understand the normative rationale that led Abū Bakr to reject *tadāwī*? Why is *tadāwī* not obligatory if the preservation of life is a fundamental normative moral duty in Islamic ethics, as we know from *maqāṣid al-Sharīʿah*? How do we reconcile the overarching duty to preserve life and the possibility of withholding or withdrawing medical treatment in EoLC, which would undoubtedly allow death to occur? (cf. Sachedina, 2009, p. 9, 20–29)

It is possible to provide some sort of rational justification for the rule on *tadāwī* and the permissibility of withholding or withdrawing medical treatment by resorting to the notion of *maṣlaḥa* (social good). A foundational proposition of Islamic legal theory is that God wills the *maṣlaḥa* of humankind. It is a proposition that commands broad consensus across different schools of Islamic theology and jurisprudence, including the traditionally rival schools of Ashʿarites and Muʿtazilites, both of which agree that God's purpose is to promote people's interests (Elmahjub, 2019, p. 318). As a conventional rule of Islamic normative analysis, it serves as the starting point for Muslim jurists – both classic (e.g. ʿIzz al-Dı̄n ibn ʿAbd al-Salām, 1991, p. 10) and modern (e.g. al-Raysuni, 2005, pp. 280–281) – who subscribe to *maqasid* and believe that rulemaking in Islamic law should primarily be informed by choices that promotes the welfare of humankind. The proposition can be interpreted in a purely practical sense as implying a duty to order good and forbid evil, or a broad ethical and normative sense, as in this article.

In the latter sense, the proposition is often understood to mean that moral choices should bring about good (*jalb al-manfaʿa*) and prevent harm (*dafʿ al-ḍarar*). This normative statement is regularly used in Islamic bioethical reasoning to justify decisions in the EoLC environment. Scholars normally engage in consequentialist analysis through empirical observation of a patient's medical conditions and cost and benefit to the public health system. It is often said that medical treatment for a moribund patient is harmful, and expectations of benefits (recovery) are extremely minimal. Accordingly, the right thing to do is to permit acts and omissions that lead to the termination of medical treatment even if this results in certain death (Sachedina, 2009, p. 9, 20–29).

However, this line of reasoning lacks depth. It does not explain why it is permissible to depart from the initial obligation to preserve life and engage in utilitarian analysis of pleasure, pain, cost, and benefit. Medical futility cases are based on a value judgement that death is highly probable and that the expected quality of life, even if the patient is saved, is not objectively satisfactory. Swetz et al. (2014, p. 954) argue that, ultimately, medical judgements remain probable, no matter how great an attempt is made to employ an evidence-based approach to determine futility. How, one may ask, can this value judgement override the foundational Islamic obligation to preserve life, especially considering that human error is also probable in many cases? Indeed, Berge et al. (2005), in their study of errors in the determination of medical futility, found that the margin of error in prognoses could be as high as 23 per cent. While it is generally assumed by those who prioritize consequentialist reasoning that the obligation to preserve human life diminishes in the EoLC, the assumption is not accompanied by adequate moral evaluation. This is the challenge which the present article will seek to address by pondering the scope of this obligation in the EoLC environment.

Islamic bioethical analysis of EoLC has a limited perception of the kind of futility that permits cessation of medical treatment. Existing deductivist or positivist reasoning does not seem to capture the perplexing uncertainties and situational complexities associated with EoLC. There is no clear vision of the essence of medical futility that would permit acts and omissions terminating a patient's life (Mohiuddin et al., 2020). Does it exclusively depend on the medical prognosis of the expected life span where it would be possible to withdraw medical treatment if doctors predict that the patient has only a short time left to live? Or does medical futility also include patients who might live for an extended period but with a low quality of life? At its core, existing Islamic reasoning on EoLC does not seem to appreciate that medical futility is a highly elusive and moving target. It seems to assume that it involves patients in vegetative states or patients for whom medical treatment has repeatedly failed (e.g. unsuccessful chemotherapy for metastatic cancer).

Moreover, Islamic bioethical literature typically focuses on a limited range of possible acts or omissions, including withdrawing or withholding ventilators, medicine, or artificial nutrition or hydration. However, medical conditions that could be classified as futile are diverse and more complex. Take, for instance, an 85-year-old patient with advanced dementia who also lives with a pacemaker. They are not on a ventilator and do not require artificial feeding. Physicians cannot provide a satisfactory prognosis for their condition, but they are difficult to treat because of their abusive behavior towards caregivers. Their immediate family members cannot afford the medical treatment and think that the patient has a low quality of life. According to the existing framework of Islamic bioethical reasoning, can the family ask for the patient's pacemaker to be turned off to hasten death? Does the rule on *tadāwī* and the harm/benefit calculus justify the family's request? What should we prioritize here: the intrinsic value of human life as mandated by the Islamic sources of moral obligation or the consequentialist cost/benefit analysis to minimize suffering and efficiently allocate medical resources to their most effective use?

## EoLC and Islamic normative ethics

The rest of this article shows that Islamic sources support introducing a broad normative vision for bioethical reasoning regarding EoLC. It engages with Islamic jurisprudence and legal theory not so much as a body of rigid sources of moral obligations but rather as an ethical discourse and a framework for identifying normative principles and justifying ethical judgements. At its core, Islamic jurisprudence and legal theory are concerned with theorizing good and evil, right and wrong, and ultimately seeking answers to the question of morally required conduct for a Muslim in a particular context. There is room for a paradigm shift in the methodology of Islamic bioethical reasoning towards a theory of Islamic bioethics that states and defends principles for morality in the context of EoLC – an approach that provides a range of rules to determine at the case level which actions are right, which actions are wrong, what is permitted, and what is forbidden.

It is important to reiterate that the proposed paradigm shift in methodology does not seek to challenge the general importance of textual sources in moral reasoning in Islamic legal theory. In the case the legal and ethical questions for which there are specific revelatory injunctions, textual sources are still the first point of reference for creating moral knowledge about the right conduct. However, textual sources have a limited scope of application when it comes to emerging challenges in human societies that were not anticipated at the time of revelation. This explains the conventional wisdom of Islamic jurisprudence *al-nusus mutanahiyya wa al-waqaeiʿ ghaiyru muntanahiyya* (texts are limited and social change is limitless). This observation is particularly true when it comes to modern bioethical challenges. Textual sources do not provide specific behavioral instructions for the evolving, complex, and vexing moral questions within EoLC in particular. EoLC is an area of moral uncertainty and, as such, it is open to value judgements. If the textual sources cannot enable a direct ethical judgement for bioethical challenges in EoLC, one must adopt an ethical approach to construct normative Islamic principles to justify a moral judgement for EoLC from an Islamic perspective. Premodern Muslim jurists overwhelmingly argued that textual sources are permeated with an overarching vision of *maṣlaḥa*, the purpose of which is to establish good and prevent harm (al-Fā sı̄, 1963, pp. 3–7, 41; al-Ghazālī, 1971, p. 483). This vision operates as a foundational bedrock for moral reasoning in Islamic law and ethics. Jurists would invoke notions of *maṣlaḥa* when interpreting and applying existing textual authorities to a particular moral question, extending the law by analogy or engaging in normative analysis to establish norms for textually unqualified issues (al-Raysuni, 2005, pp. 280–281). I aim to develop a unique vision of *maṣlaḥa* as a theory of good, ethical value, and right conduct. I view *maṣlaḥa* as a normative construct aimed at achieving a moral state of affairs compatible with the divine will, a methodology, and a substantive body of norms, connecting divine intentions, as understood through textual sources, to human good.

The vision of *maṣlaḥa* I introduce in this article differs from that normally debated in modern literature on Islamic theory. Modern Islamic studies tend to equate *maṣlaḥa* with notions like public interest or public welfare, regarding it as a utilitarian concept that aims to minimize harm and maximize good for the greatest number. As I argue elsewhere (Elmahjub, 2021, p. 7), this understanding of *maṣlaḥa* does not capture its technical complexity when used as a term of art in Islamic jurisprudence, which is much broader.

How, then, is this broad understanding of *maṣlaḥa* relevant to EoLC issues? The locus of analysis in EoLC is human life. *Maṣlaḥa,* as a theory of good, can provide us with moral insights on the ethical value of human life and how we should formulate normative positions to guide human action when human life is at stake. If the Islamic system of ethics, through its sources of moral obligations, instructs Muslims to seek *maṣlaḥa*, what is the essence of *maṣlaḥa* that we are trying to protect and promote in the EoLC environment? How do we discover it?

As far as the ethical value of human life is concerned, Muslim jurists unanimously agree that preserving human life is an overarching objective of Islamic systems of law and ethics. Human life is intrinsically valuable, and the right conduct lies in preserving life and protecting it against life-threatening dangers. However, there is disagreement about the content that we can give to this expression. My reading of existing Islamic jurisprudence shows that there are two approaches to the normative ethics of human life. There is a deontological approach that views human life as sacred and valuable in its own right. This approach imposes a categorical duty to preserve life regardless of the consequences. The other approach is consequentialist. While it accepts the central value of human life, it determines that the right conduct is that which promotes an optimal state of affairs, even if this leads to sacrificing human life in certain situations.

In Sections 4.1 and 4.2, I show how these two approaches could assist in identifying, justifying, and balancing principles for Islamic bioethical reasoning in EoLC. Ultimately, I suggest that the right approach to EoLC ethics lies in a framework that reconciles these two ethical approaches. I call the proposed approach “Islamic evaluator relativity.” This approach places Islamic deontology of the ethical value of life as a first-order principle and the rival consequentialist approach as a second-order principle. It then moves to show that the identities of evaluators in the EoLC environment (physicians, patients, and families) should be taken into consideration when balancing options to determine the moral choices in the Islamic worldview.

### The Islamic deontological approach to EoLC

Deontological normativity holds that what makes conduct morally right is its conformity with preexisting moral norms. A deontologist would argue that each human life is intrinsically valuable as an end in itself. On the normative side, moral agents must obey ethical duties to preserve the intrinsic value of human life. Generally, no other moral data external to the value of life is relevant in determining morally required choices. This includes the quality of human life itself, pain and pleasure, or cost and benefit. Actions not in accord with the intrinsic value of life may not be performed, regardless of our expectations of harmful consequences. In comparative moral philosophy, the central figure of deontological moral theory is Immanuel Kant. He advocates the categorical imperative, which states that it is morally blameworthy for moral agents to treat human life as a means for achieving other purposes. Based on this ethical proposition, a person may not engage (or require others to engage) in acts or omissions to terminate their life *in order to* end suffering or save costs. Life here has no instrumental value to serve other purposes (Kant, 1785/1996).

In the Islamic sources of moral obligation, textual norms consistently emphasize the central ethical value of human life. Its protection is highly praised, while its unlawful termination carries the most severe punishment (an eternity in hellfire) (*Qurʾān*, 5:32, 4:92). In normative terms, it is possible to extract from Islamic jurisprudence a trend that formulates the ethical value of human life in a basic deontological vision. I locate this vision in the writings of Imām al-Ḥaramayn al-Juwaynī (d. 1085) and Abū Ḥāmid al-Ghazālī (d. 1111). Both jurists understood Islamic moral reasoning as some form of “textual deontology” where revelatory norms act as an exclusive source for ethical value. The right conduct becomes that which accords with a preexisting norm sanctioned by textual sources. In this model, rational, consequentialist, and utilitarian calculations of harm and benefit play no role in norm creation. According to al-Juwaynī, the content of ethical value depends solely on the ethics of revelation. Good (*ḥasan*) and evil (*qabīḥ*) do not depend on some intuitionist account of moral knowledge that opens the door for extrascriptural reasoning (al-Juwaynī, *al-Burhān fī usụ̄ l al-fiqh*, 1980, p. 91). He was probably the first to define *maṣlaḥa* as that which revelation intends to achieve (*maqṣūd al-sharʿ*) (p. 48). The scripture is the guide when proclaiming something to be part of the divine scheme as a good that must be promoted or an evil that must be prohibited. The good is that which God declared as good and provided a reward for doing; the evil is that which God declared as evil and ordained punishment for doing (al-Malik, *al-Burhān fī usụ̄ l al-fiqh*, pp. 1–10).

Al-Ghazālī shared al-Juwaynī's vision in describing ethical value as *maqṣūd al-sharʿ*. According to al-Ghazālī, revelation promotes an overarching ethical and normative vision agreeable to the interests of humankind (*munāsiba*). Revelation is designed to enable moral agents (*mukallafun*) to bring about good and avert evil (al-Ghazālī, *Shifāʾ al-ghalīl*, p. 221). However, good and evil, as well as benefit and harm, do not depend on human perception, but can be known through textually inspired inductive and deductive surveys. Al-Ghazālī's taxonomy of ethical value identified five primary objectives: the promotion of religion, human life, lineage, intellect, and wealth (*al-Mustaṣfá*, n.d., pp. 481–482). The ethical value of human life is my concern here. In unmistakeably deontological terms, al-Ghazālī asserts that human life is sacred in its own right. If we have to make a moral choice between (a) respecting our moral duty to individual life or (b) engaging in consequentialist moral analysis to sacrifice it to save a few lives, al-Ghazālī would err on the side of the duty to human life, not the standard utilitarian calculus of achieving the greatest good for the greatest number. This is why he declared as impermissible the act of throwing one passenger from a sinking boat to save the majority. According to al-Ghazālī, the life of that one person is sacred and cannot be sacrificed to the good of the other passengers (p. 489). Although some scholars have sought to explain al-Ghazālī's normative theory in utilitarian terms, notably by focusing on his famous shield example where he stated that it was permissible to kill a few innocent captives used as a human shield if this was the only available option to save a Muslim land and community from invasion (Nusseibeh, 2017, p. 89), there is growing opposition in comparative Islamic studies to this view of al-Ghazali's normative ethics (Elmahjub, 2021, p. 5; Emon, 2005;.Koujah, 2019, p. 136). In sum, the duty to preserve human life takes priority over any consequentialist calculations of cost and benefit, or, as a deontologist would say, the right precedes the good.

What should we make of this deontological Islamic vision of human life when applied to EoLC? The answer depends on how we answer another question: Does the life of a terminally ill person have a moral existence in the Islamic worldview? The answer is a resounding yes! If we set aside scenarios of brain death where Muslim scholars doubt the existence of life itself, the life of a terminally ill person still exists. This is true even if some organ functions depend on external medical support. In this sense, the duty to respect human life does not diminish. This explains why Muslim jurists and institutions have refrained from engaging in consequentialist reasoning about euthanasia and assisted suicide. Considerations of pain and suffering, harm and benefit, and cost to the healthcare system cannot justify a deliberate, intentional act of a physician or the patient to end their life. In this context, Muhammad al-Ṭāhir ibn ʿĀshūr seems to understand the promotion of life in deontological terms, even if he does not use this term specifically. He contends that it is imperative to protect the life of a poor and sick person suffering from an incurable disease, regardless of the consequences. The value of human life itself cannot be subjected to a cost/benefit or pain/pleasure analysis (al-Ṭāhir ibn ʿĀshūr, 2001, p. 98, 329).

The deontological vision towards the ethical value of human life continues to be binding in EoLC. It imposes a duty on healthcare providers to spare no effort to prevent premature death. This vision also gives the patient the right to receive adequate, reasonable, and appropriate medical treatment. However, it does not transform the obligations of all moral agents in the EoLC context into an absolute moral duty to provide extraordinary, onerous, intrusive, or futile medical treatment. The duty to preserve life does not necessarily become a duty to save life in all circumstances and at all cost.

The scope of the duty to preserve life is limited to the best possible performance, not to guarantee results (i.e. keeping patients alive). A physician discharges their duty to protect life if they takes adequate actions to demonstrate the required respect for the sanctity of human life. This should not include providing heroic medical treatment to medically futile cases. Here, we should distinguish between acts and omissions made intending to precipitate death and acts or omissions aimed at removing an impediment. We may not absolutize the Islamic duty to preserve life and transform it into an obligation to heal futile cases and keep patients indefinitely on life-sustaining means. It is hard to accept that the Islamic system of ethics considers as *maṣlaḥa* the possibility of keeping a terminally ill patient alive even if this leads to a hardship that is inhumanely imposed. Death is a natural feature of the Islamic worldview. The Qurʾān recognizes this fact and does not depict death as an evil to be avoided in all circumstances (*Qurʾān*, 3:185, 67:2, 62:8). This probably explains al-Ghazālī's position towards medical treatment for moribund persons. Despite his firm emphasis on a duty-based approach to the sanctity of human life, he was prepared to accept that moribund patients have a legitimate right to refuse medical treatment and allow death to take its natural course (*Iḥyāʾ ʿulūm al-dīn*, n.d., p. 287). In this context, the deontological vision of Islamic ethics on human life does not necessarily entail categorical prohibitions against withdrawing or withholding medical treatment if rigorously established medical opinions determine that treatment is futile and the patient is terminally ill.

To sum up, there is a prima facie general normative principle in Islamic ethics that mandates the preservation of life. This principle remains operational in the EoLC environment unless and until additional moral data about the human life in question enters our field of ethical evaluations. The general bindingness of the duty to preserve life may well be eclipsed by the countervailing need to avoid futile treatment.

### The Islamic consequentialist approach to EoLC

Deontology is not the only approach that we can see in Islamic ethics. It is possible to view moral reasoning in Islamic jurisprudence and legal theory as a crude form of consequentialist or utilitarian approach to ethics that seeks to maximize value in the EoLC environment. In this sense, moral reasoning moves away from assuming a preformed categorical duty towards preserving individual human life: human life is not always treated as an end in itself. Still, it could be assessed in an instrumentalist way. The right thing to do becomes that which achieves an average good state of affairs that minimizes harm, pain, and inefficient allocation of resources and maximizes benefit, happiness, and the overall good for the majority, even if this leads to sacrificing human life. The general idea of maximizing good and minimizing evil is central to Islamic normative thought. There is a growing trend in comparative Islamic studies that explicitly associates *maṣlaḥa* in Islamic theology and jurisprudence with a consequentialist theory of morality, the main purpose of which is to promote the average good in the same way as in standard consequentialist or utilitarian theories (Hourani, 1960, p. 273; March, 2009, p. 63, for whom “conceptions of *maṣlaḥa* are the greatest single example of consequentialist-utilitarian reasoning”). We could extract general consequentialist or utilitarian methodologies from a great number of Muslim jurists (Hallaq, 2009, p. 116; Hallaq, 1997, p. 42, 224; Kerr, 1966, p. 114, 121). However, a few leading examples will suffice, since jurists share the basic consequentialist idea that identifies the intrinsically valuable “Good” and seeks choices to increase it. I focus here on Fakhr al-Dīn al-Rāzī (d. 1210), al-ʿIzz ibn ʿAbd al-Salām (d. 1261), and Imām al-Shātịbī (d. 1388) and highlight how their consequentialist methodologies would affect EoLC ethics.

For a moral methodology to be branded consequentialist it must be built on two affirmations. First, the metaethical thesis establishes some form of intrinsic ethical value (normally called “the Good”). Second, a normative proposition that suggests that morality lies in making choices to maximize that “Good.” If we dismantle the methodologies of al-Rāzī, al-ʿIzz, and al-Shātịbī in relation to moral reasoning, we will be able to locate these two affirmations in their contributions.

In *al-Maḥṣūl fī ʿilm uṣūl al-fiqh*, al-Rāzī seems keen to affirm the standard Ashʿarite metaethical claim on ethical value. Our moral knowledge of *ḥasan* (good) and *qabīḥ* (evil), al-Rāzī asserts, must exclusively be derived from revelation (*al-Maḥṣūl*, 1988, Vol. 1 pp. 108, 123). Following the footsteps of al-Juwaynī and al-Ghazālī, he maintains that Muslims need to trace textual commands and prohibitions to form their view on what we would today call ethical value. In this sense, he does not depart from al-Juwaynī's definition of goodness as the purpose of revelation (*maqṣūd al-sharʿ*). Moreover, he not only understood *maqṣūd al-sharʿ* through al-Ghazālī's taxonomy of the five basic social goods but also copied some of al-Ghazālī's practical examples as introduced in *al-Mustaṣfá* (al-Ghazālī's main work on legal theory) (al-Rāzī, *al-Maḥṣūl*, 1988, Vol. 5 pp. 160–162).

However, al-Rāzī shifts sharply from the standard Ashʿarite position in his explanation of the content and scope of ethical value. According to al-Rāzī, revelatory norms found in textual sources are associated with design principles agreeable to human nature (*munāsiba*). He contends that the ethical value intended by God is built on ensuring that all moral agents secure benefit (*manfaʿa*) and avoid harm (*mafsada*). Al-Rāzī then reduces the ethical value to a form of rational/hedonic calculation by assimilating *manfaʿa* with pleasure (*ladhdah*) and *mafsada* with pain (*alam*). According to al-Rāzī, pleasure and pain need no further explanation since both are connected to the individual's sensations (*al-Maḥṣūl*, 1988, Vol. 5 p. 158). In other words, good and evil are ultimately discoverable through ontological reasoning as perceived by the human senses. This rational view of ethical value contrasts with al-Juwaynī's and al-Ghazālī's belief in the exclusively textual (and revelatory) source of ethical value and can be assumed to have been inspired by Ancient Greek notions of the hedonic nature of value (Haywood, 1979, p. 266), especially in view of al-Rāzī's claims about the role of pain and pleasure as motivating factors for human actions (*al-Mabāhith*, n.d., pp. 388–397). Our perception of Al-Rāzī's consequentialist/utilitarian approach is consolidated by his normative claims. After determining that ethical value is built on a pain/pleasure calculus, he suggests that the right action depends on a consequentialist weighing of human conduct. If an action brings more good than evil, it becomes imperative, and vice versa (*al-Maḥṣūl*, 1988, Vol. 5, p. 278; Vol. 6, p. 166).

Similar to al-Rāzī, the overall structure of al-ʿIzz's formula on ethical value and its normative implications supports the consequentialist/utilitarian approach to moral reasoning in Islamic ethics. Al-ʿIzz also makes a metaethical claim about the existence of an intrinsic good as well as a normative claim that the morally required choice is to maximize that good. He begins his discussion on ethical value by insisting that human reason has an ontological capacity to determine the content of mundane interests. Humans are naturally disposed to realize the difference between good and evil. For instance, empirical observation shows that human beings intuitively acknowledge the sanctity of life, property, and honor (*al-Qawāʿid al-kubrā*, p. 9).

Al-ʿIzz defines the manifestations of ethical value by declaring that the essence of the good is pleasure and happiness, and the essence of evil is pain and sadness (p. 15). He then draws attention to the complex structure of ethical value by maintaining that pure good and pure evil are rare. Typically, moral reasoning entails degrees of goodness and evilness. Al-ʿIzz's approach to the normative analysis of value is similar to al-Rāzī in holding that the morally required choice depends on a consequentialist weighing of good and evil (p. 12). The right thing to do is to maximize the good (i.e. happiness) and minimize evil (i.e. sadness). However, unlike al-Rāzī, there are different forms of normative positions that we can attach to human actions depending on the relevant degrees of good and evil (p. 15). We cannot reduce moral judgements to mere obligatory acts and omissions. The classic divisions of moral judgement (*taqsimāt al-ḥukm al-sharʿi*) in Islamic legal theory should inform the normative implications that we can attach to ethical value. For instance, pure good leads to obligatory action, pure evil to prohibition, equal expectations of good and evil to permissibility, and so on (pp. 12, 267 *et seq.*).

How might this consequentialist approach apply to Islamic bioethical reasoning in EoLC? It should be noted that al-Rāzī and al-ʿIzz, like al-Juwaynī and al-Ghazālī, believe that preserving human life is a textually mandated objective in Islamic ethics. Therefore, their consequentialism would not open the door to a free-standing utilitarian analysis of EoLC issues. For example, al-Rāzī and al-ʿIzz's model would not permit a departure from the absolute prohibition on euthanasia or physician-assisted suicide to a position whereby these acts are permitted if they lead to reduced pain and suffering and to maximizing the efficient allocation of health resources. However, apart from prohibitions against intentional killings of any sort, their model enables evaluators to seek an overall state of affairs that maximizes pleasure and happiness and minimizes harm and pain, even if, in some cases, this leads to the inevitable outcome of sacrificing individual life. This understanding fits perfectly with al-Shātịbī's vision of Islamic consequentialism. He states that:

Instructing humankind to tally the consequences of their actions (*ma’ālāt al-’afʿal*) is an intended objective of revelation. A jurist (*mujtahid*) will not arrive at a moral judgement on acts and omissions without giving due consideration to the expected consequences of those acts and omissions. (*al-Muwāfaqāt*, n.d., Vol. 5, p. 177)

Al-Shātịbī applies his formulae of consequentialist reasoning to several examples throughout his book, *al-Muwāfaqāt* (Vol. 2, p. 27). For instance, he maintains that if we face a situation where we need to decide to choose an action that promotes the good of the majority compared to the interest of the few, we should err on the side of the majority. Accordingly, for al-Shātịbī, the right thing to do is to sacrifice one human life to save the majority (Vol. 2, p. 64). Obviously, al-Shātịbī's reasoning endorses the standard utilitarian position in the famous trolley dilemma: the right thing to do would be to take the action that leads to one person being killed and the rest being saved! We can contrast this position with al-Ghazālī's deontology towards human life where the exact opposite outcome would be achieved (i.e. categorical prohibition to sacrifice life even if the intention is to save many lives).

General consequentialist reasoning seems to be quite intuitive in mainstream bioethical discourse. We can find its clearest manifestation in the broad normative principles of nonmaleficence and beneficence espoused by Childress and Beauchamp. Consequentialism would be the philosophical parent of these two principles as both are designed to justify choices that lead to avoiding the causation of harm (nonmaleficence) and promoting the optimal balance of benefits against risks and costs (beneficence) (Beauchamp & Childress, 2008, p. 12). Moreover, consequentialist reasoning is at the heart of analytical frameworks that justify medical decisions and policies aiming to maximize the efficient allocation of scarce health resources (Cohen, 1996, pp. 267–272; McKie et al., 1998). EoLC decisions that are made according to these principles and frameworks focus mainly on promoting choices that would reduce pain and suffering and maximize efficient use of healthcare resources. A deontological duty towards life does not usually feature as a prominent consideration.

The intuitive appeal of consequentialist reasoning meant that it made its way into Islamic bioethical discourse. The principles of beneficence and nonmaleficence are typically associated with central Islamic maxims such as *lā ḍarar wa-lā ḍirār* (no harm shall be inflicted or reciprocated) and “No harm, no harassment” (Sachedina, 2009, p. 47). Islamic institutions that deal with bioethical issues justify their decisions by resorting to consequentialist calculations of benefit and harm and permitting options that maximize benefit for the majority. Within this normative environment, the good and right do not necessarily mean preserving life at all costs. The aim should be to minimize harm and suffering and maximize cost savings even if this leads to forgoing life-sustaining medical treatment (pp. 47, 169–170).

Consequentialist reasoning is essential for the optimal structure of normative Islamic bioethics. In any given moral situation we face in EoLC, we simply cannot ignore the influence of pain, suffering, and dignity of patients, scarce healthcare resources, and financial and emotional cost to families. We cannot account for these essential considerations without a consequentialist approach to morality. Moral agents in EoLC are not trying to justify decisions to sacrifice human life in ordinary circumstances, nor are they actively seeking artificial inducement of death-hastening conditions. The subject matter for moral interrogations is human life in a terminal stage that is normally subject to constant hardship. Those who would defend the categorical/deontological duty to preserve life might argue that we can sustain life through palliative care (PC) options. Advances in medical sciences enable healthcare providers to prolong the lives of terminally ill patients and alleviate their pain. Thus, our duty should be to use all available means to preserve life. However, PC availability does not necessarily transform our duty to preserve life into an absolute moral norm. PC is not a life-saving process. Accordingly, it would be misleading to suggest that PC can maintain a normal life. In fact, PC itself can produce additional hardships for a dying person. Patients normally undergo trial and error to get optimal PC, and this may lead to additional harm and suffering.

Moreover, the best available PC does not guarantee complete relief from all pain and suffering. On the contrary, it could impose additional distress on patients in the form of loss of awareness due to sedation or side effects including incontinence and nausea. It is difficult to accept that this significant hardship is compatible with the Islamic normative vision and overall system of ethics, which makes averting hardship an organizing objective for all Islamic norms.

## Islamic bioethical relativism

The ethical dilemmas of EoLC are complex and defy formulaic applications of given rules to given issues. Therefore, it is essential not to restrict our mode of reasoning to a positivist vision oriented towards looking for specific behavioral instructions and then applying them across the dynamic range of ethical uncertainties in EoLC. As discussed above, Islamic textual sources and jurisprudence provide the basis for an alternative normative vision to address the challenges that we see in EoLC. This alternative normative vision can help us identify and justify moral norms, weigh them against each other, determine their priority, and ultimately apply them to achieve an optimal state of affairs that reflects our intuition of the divine command and human good. If we agree that it is undesirable to absolutize Islamic bioethical reasoning through positivist approaches, the appropriate alternative would be to relativize it through contextual and normative reasoning. Islamic bioethical relativism could influence our thinking about the substantive normative principles of bioethical reasoning as well as the identities of the evaluators/moral agents and their motives. Islamic relativism manifests itself in meshing together the deontological and consequentialist frameworks discussed above and taking note of the relevance of the evaluators’ identities in determining the right conduct.

When we consider Islamic jurisprudence as an ethical enterprise, there is no good religious or theoretical reason to think of it as an absolute system of ethics. We are not compelled to accept some philosophical orthodoxy that would lead us to choose between either deontological or consequentialist normativity to justify Islamic ethical value and guide human action. The Islamic system of ethics is best understood as a hybrid system combining both consequentialist and deontological moral positions applicable to any given ethical context (Elmahjub, 2021, p. 27; Moosa, 2014, p. 36). EoLC is an ideal object for this hybrid vision of Islamic ethics. A hybrid theoretical structure that combines various elements of consequentialism and deontology would not be unique to Islamic ethics. It is an essential feature of mainstream bioethical discourse that we can easily locate in the four principles of bioethics. In clear terms, Childress associated himself with Christian deontology while Beauchamp identified himself as a rule utilitarian (1979, p. 40).

We can think of the Islamic deontological position towards human life as a first-order principle. As mentioned above, in EoLC settings we are still dealing with human life that has continuous moral existence. Medical diagnoses of terminal illness and futility is based on a probable value judgement that does not change this biological fact and ethical status. Accordingly, Islamic deontic normativity justifies the necessary constraints to reject any active or intentional intervention to end life through euthanasia or physician-assisted suicide. It also mandates obligatory safeguards to prevent premature medical decisions to withhold or withdraw medical treatment. Evaluators’ behavior is guided to give the utmost respect to the patient's right to life. This could be behaviorally reflected in different ways. For example, we could require unanimous agreement from a multiprofessional team indicating that the patient is in fact “dying.” We could also require a minimum period to pass between the decision to stop medical treatment and its fulfilment. Finally, we could require expanding the circle of consultations to include patients and surrogate decision makers. These first-order deontological constraints will be necessary to avoid a quick shift to the end-of-life pathway that appeals to the pragmatism of consequentialist analyses of harm/benefit and resource allocation. An Islamic bioethical approach to EoLC will not allow consequentialism to operate as a first-order principle to override respect for human life – this can mean tolerating hasty decisions that lead to death. This is particularly worrying for ageing persons, new-borns with disabilities, and persons from a low socioeconomic background (Beauchamp & Childress, 2008, pp. 145–154).

Shifting to a consequentialist/utilitarian methodology would be acceptable if those involved in assessing the ethical status of EoLC demonstrated adherence to the said first-order deontological principle. A blanket adoption of deontology is impractical and indefensible. Only very few extraordinary persons will be able to continue medical treatment for futile cases against all objective medical assessment of terminal illness. This is not necessarily the morally required approach in Islamic ethics. Accordingly, evaluators should be able to extend their analysis to relevant moral data concerning pain, suffering, prospects of recovery, quality of life, and costs to families and healthcare resources. In some cases, it can never be wrong to opt for moral choices that produce an optimal state of affairs, even if this leads to removing impediments so that natural death can take place.

Finally, we should not ignore the identities of the evaluators as a relevant factor in determining the moral status of Islamic moral judgements in EoLC. This is particularly true for physicians. At its core, EoLC ethics depends on value judgements combining medical expectations and the intuitions of evaluators and their perception of each individual case. It makes sense to relativize moral judgements concerning EoLC to the evaluator's position. An optimal Islamic framework for EoLC bioethics will take into consideration evaluator relativity. Since principal evaluators in EoLC are physicians, our default assumption about physicians is that they are competent moral judges of medical diagnosis and prognosis. They are reasonable, intelligent, informed, and empathetic. They value human life, do not judge prematurely to serve their self-interest, and are unlikely to allow their emotions to cloud their judgements. In Islamic ethics, praiseworthiness and blameworthiness of judgements are significantly tied to agents’ intentions and motives (al-Nawawī, 1999–2020). If we take into consideration these assumed traits of physicians as competent moral judges, we should be able to afford them the necessary latitude to exercise their value judgements. In any case, those who pursue Islamic moral judgements in areas of moral uncertainty such as EoLC will not be morally blameworthy according to Islamic ethical norms, so long as they did their best to ensure that their judgement reflected the divine will. When moral choices are formulated as value judgements (*amr ijtihādi*), the famous Islamic maxim applies so that the evaluator (*mujtahid*) will be praiseworthy regardless of the choices they make (Ṣaḥīḥ al-Bukhārī, Book 96, Hadīth 79, n.d.).

## Limitations

This research is the first to offer a novel hybrid normative approach to determine the right conduct in matters of EoLC. However, it is not without limitations. In presenting the Islamic worldview on EoLC, the study focuses on leading figures of classic Sunni schools of Islamic jurisprudence. It does not include inputs from Shia jurisprudence or modern reform figures such as Muhammad Abduh and Rashid Rida, as their inclusion would have extended the scope of the article beyond reasonable limits. Additionally, this study does not have an empirical component. It would have been helpful to include an empirical study to examine how Muslim physicians, patients, and their families make decisions in connection with EoLC. In particular, does the distinction between Islamic deontological and consequentialist normativity towards human life presented in this article have any practical merit in the decision-making process?

## Conclusion

An optimal framework for Islamic bioethics in EoLC will consider the critical evaluation of existing positivist methodologies. For instance, it is not enough to rely on a posited rule stating that *tadāwī* is not obligatory and apply this rule to the complicated ethical situations of the EoLC environment. Also, more should be done to explain why it is possible, from an Islamic perspective, to skip the discussion on the sanctity of life as an overarching objective of Islamic morality and view EoLC through the prism of the consequentialist calculation of harm and benefit. There is room to develop a broad Islamic ethical framework to determine the good and the right conduct in EoLC. To do so, we need to start thinking of *maṣlaḥa* as a normative construct and ask, What is the *maṣlaḥa* that we are aiming to protect in EoLC? Human life is the *locus classicus* of EoLC ethics. This article argues that, as far as human life is concerned, there are at least two options for expressing and determining *maṣlaḥa* in the EoLC context. First, we have a deontological approach that would lead us to demonstrate unconditional respect for the intrinsic value of human life. On the other hand, we have a consequentialist approach that would enable us to tally considerations of pain, harm, costs, and risks. These two approaches are not necessarily mutually exclusive. The right approach to Islamic EoLC bioethics may be found in some hybrid version that combines Islamic deontological and consequentialist thinking towards the moral value of human life.

Additionally, it is essential to bring into clearer focus the character and differential particularity of evaluators in Islamic EoLC bioethics. Evaluators in EoLC are competent moral judges. So long as there are adequate safeguards to avoid premature medical decisions of futility and the end of medical treatment, we should afford medical professionals and families the moral discretion to determine the right thing to do. This is particularly true if we have no good reason to doubt the purity of their intentions towards the dying person. Evaluators are not required to seek Islamic ethical justification in any kind of strict harmony between the transcendent realm of preformed ethical norms and moral judgements that reflect divine commands. Rather, they can form a dialectical relationship between Islamic normative principles of deontology and consequentialism. Ultimately, they could seek judgements in the overall meshing of these principles and their intuitions about individual cases.

## References

[CIT0001] al-Bagha, M. (n.d.). *Athar al-Adillah al-Mukhtalaf fīha fī al-fiqh al-islāmī*. Dār al-Imām al-Bukhārī.

[CIT0002] al-Bār, M. (2015, May 10–13). *al-Tadāwī qurb nihāyat al-ḥayāh wa al-inʿāsh al-qalbī al-riʾawī*. [Paper presentation]. Islamic Fiqh Academy, 22nd Session.

[CIT0003] al- Bār, Muhammad Ali (1995). *Ahkâm al-Tadāwī*. Dār al-Manāra.

[CIT0004] al-Fāsı̄ʿ, A. (1963). *Maqā sịd al-Sharı̄ ʿa al-Islamiyya wa-Manakibuha [The objectives of Islamic Shari’a and their importance]*. Maktabat al-Wahda al-ʿArabiyya.

[CIT0005] al-Ghazālī, A. H. (1971). *Shifāʾ al-ghalīl (Ḥamd ʿUbayd al-Kubaysī, Ed.)*. Matḅaʿat al-Irshād.

[CIT0006] al-Ghazālī, A. H. (n.d.). *al-Mustaṣfá (Ḥamza b. Zuhayr Ḥāfiẓ, Ed., Vol. 2)*. Sharikat al-Madīna al-Munawwara lil-Ṭibāʿa.

[CIT0007] al-Ghazālī, A. H. (n.d.). *Iḥyāʾ ʿulūm al-dīn (Vol. 4)*. Dār al-Maʿrifa.

[CIT0008] al-Jufaiyyrī, M. (2008). ʿAlī al-Mawt al-Rahīm min Mandhūr insānī wa Islāmī [paper presentation]. 6th Doha conference on interfaith dialogue.

[CIT0009] al-Juwaynī. (1980). *al-Burhān fī usụ̄ l al-fiqh (ʿAbd al-ʿAzị̄ m al-Dīb, Ed.)*. Dār al-Ansạ̄r.

[CIT0010] al-Nawawī. (1999–2020). [Hadīth No.] 1: Actions are by intention. al-Nawawī’s Forty Hadīth. https://www.nawawis40hadith.com/nw/hadith/1/actions-are-by-intention.

[CIT0011] al-Qaraḍāwī, Y. (2013, May 20). Qatl al-Raḥma Ḥakikatuhu wa Ḥukmuhu. https://al-qaradawi.net/node/4108.

[CIT0012] al-Raysuni, A. (2005). *Imam al-Shātịbī‘s theory of the higher objectives and intents of Islamic Law*. International Institute of Islamic Thought.

[CIT0013] al-Rāzī, Fakhr al-Dīn. (n.d.). *al-Mabāhith al-mashriqiyya fī ʿilm al-ilāhiyyāt wa-al-tabiʿiyyāt (Vol. 1)*. Markaz taḥqiqā Kampiyutiri- ʿUlumi Islāmi.

[CIT0014] al-Rāzī, Muhammad Fakhr al-Dīn. (1988). *al-Maḥṣūl fī ʿilm uṣūl al-fiqh (vols. 1, 5, 6)*. Dār al-Kutub al-ʿIlmiyya.

[CIT0015] al-Ṭāhir ibn ʿĀshūr, M. (2001). *Maqāṣid al-Sharīʿah al-Islāmiyya*. Dār al-Nafā’is.

[CIT0016] Austin, J. (1995). *The province of jurisprudence determined (Wilfrid E. Rumble, Ed.)*. Cambridge University Press. (Original work published 1832).

[CIT0017] Beauchamp, T. L., & Childress, J. F. (1979). *Principles of biomedical ethics (1st ed.)*. Oxford University Press.

[CIT0018] Beauchamp, T. L., & Childress, J. F. (2008). *Principles of biomedical ethics (6th ed.)*. Oxford University Press.

[CIT0019] Berge, K. H., Maiers, D. R., & Schreiner, D. P. (2005). Resource utilization and outcome in gravely ill intensive care unit patients with predicted in-hospital mortality rates of 95% or higher by APACHE III scores: The relationship with physician and family expectations. *Mayo Clinical Proceedings*, *80*(2), 166–173. 10.4065/80.2.166 15704770

[CIT0020] Cohen, J. (1996). Preferences, needs and QALYs. *Journal of Medical Ethics*, *22*(5), 267–272. 10.1136/jme.22.5.267 8910777 PMC1377057

[CIT0021] Daudt, H. M., Van Mossel, C., & Scott, S. J. (2013). Enhancing the scoping study methodology: A large, interprofessional team’s experience with Arksey and O’Malley’s framework. *BMC Medical Research Methodology*, *13*(1), 48. 10.1186/1471-2288-13-48 23522333 PMC3614526

[CIT0022] De Haan, J. (2002). The ethics of euthanasia: Advocates’ perspectives. *Bioethics*, *16*(2), 154–172. 10.1111/1467-8519.00276 12083156

[CIT0023] Dreier, J. (1993). Structures of normative theories. *The Monist*, *76*(1), 22–40. 10.5840/monist19937616

[CIT0024] Elmahjub, E. (2019). Transformative vision of Islamic jurisprudence and the pursuit of common ground for the social good in pluralist societies. *Asian Journal of Comparative Law*, *14*(2), 305–335. 10.1017/asjcl.2019.34

[CIT0025] Elmahjub, E. (2021). Islamic jurisprudence as an ethical discourse: An enquiry into the nature of moral reasoning in Islamic legal theory. *Oxford Journal of Law and Religion*, *10*(1), 16–42. 10.1093/ojlr/rwaa023

[CIT0026] Emon, A. M. (2005). Natural law and natural rights in Islamic law. *Journal of Law and Religion*, *20*(2), 351–395. 10.2307/4144668

[CIT0027] Ghaly, M., Diamond, R. R., El-Akoum, M., & Hassan, A. (2018). *Palliative care and Islamic ethics: Exploring key issues and best practices (special report in collaboration with research center for Islamic legislation and ethics)*. Qatar Foundation; World Innovation Summit for Health.

[CIT0028] Gorsuch, N. M. (2000). The right to assisted suicide and euthanasia. *Harvard Journal of Law & Public Policy*, *23*(3), 599–710. 12524693

[CIT0029] Hallaq, W. B. (1997). *A history of Islamic law and legal theories*. Cambridge University Press.

[CIT0030] Hallaq, W. B. (2009). *An introduction to Islamic law*. Cambridge University Press.

[CIT0031] Haywood, J. A. (1979). Faḵhr al-Dīn al-Rāzī’s contribution to ideas of ultimate reality and meaning. *Ultimate Reality and Meaning*, *2*(4), 264–291. 10.3138/uram.2.4.264

[CIT0032] Hourani, G. F. (1960). Two theories of value in medieval Islam. *The Muslim World*, *50*(4), 269–278. 10.1111/j.1478-1913.1960.tb01091.x

[CIT0033] Ibrāhīm b, Mūsa al-Shātịbī (n.d.). *al-Muwāfaqāt fī usụ̄ l al-Sharīʿa (ʿAbdallāh Darāz, Ed., Vols. 2, 5)*. Dār al-Fikr al-ʿArabī.

[CIT0034] Islamic Organization for Medical Sciences. (1981, January). Islamic code of medical ethics, Kuwait document. First international conference on islamic medicine, Kuwait Rabi I.

[CIT0035] ʿIzz al-Dı̄n ibn ʿAbd al-Salām, A. M. (1991). *Qawā ʿid al-ahḳām fı̄ masạ̄liḥ al-anām [The rules of lawmaking in the pursuit of the common good] (Ṭāhā ʿAbd al-Raʾūf Saʿd, Ed.)*. Maktabat al-Kulliyyā t al-Azhariyya.

[CIT0036] Jabbūrī. (2015, May 10–13). *ʿAbd Allāh al. Īqāf al-ʿilāj ʿan al-marīḍ al-mayʿūs min burʾih. [paper presentation]*, 22nd Session. Islamic Fiqh Academy.

[CIT0037] Kant, I. (1996). *Metaphysics of morals Mary Gregor, Trans.)*. Cambridge University Press. (Original work published 1785).

[CIT0038] Kerr, M. (1966). *Islamic reform: The political and legal theories of Muhammad ʿAbduh and Rashīd Riḍá*. University of California Press.

[CIT0039] Koujah, R. (2019). *Maṣlaḥa* as a normative claim of Islamic jurisprudence: The legal philosophy of al-ʿIzz b. ʿAbd al-Salām. In S. Siddiqui (Ed.), *Locating the Sharīʿa legal fluidity in theory, history and practice* (pp. 127–150). Brill.

[CIT0040] March, A. F. (2009). Sources of moral obligation to non-Muslims in the “jurisprudence of Muslim minorities” (*Fiqh al-aqalliyyāt*) discourse. *Islamic Law and Society*, *16*(1), 39–94. 10.1163/156851908X413757

[CIT0041] McGee, A. (2011). Ending the life of the act/omission dispute: Causation in withholding and withdrawing life-sustaining measures. *Legal Studies*, *31*(3), 467–491. 10.1111/j.1748-121X.2011.00193.x

[CIT0042] McKie, J., Richardson, J., & Kuhse, H. (1998). *The allocation of health care resources: An ethical evaluation of the “QALY” approach*. Ashgate.

[CIT0043] Mohiuddin, A., Suleman, M., Rasheed, S., & Padela, A. I. (2020). When can muslims withdraw or withhold life support? A narrative review of Islamic juridical rulings. *Global Bioethics*, *31*(1), 29–46. 10.1080/11287462.2020.1736243 32284707 PMC7144300

[CIT0044] Moosa, E. (2014). Ethical landscape: Laws, norms, and morality. In J. T. Kenney, & E. Moosa (Eds.), *Islam in the modern world* (pp. 35–55). Routledge.

[CIT0045] Muḥammad b, I. a.-B. (n.d.). Ṣaḥīḥ al-Bukhārī 7352, Book 96, Hadith 79 (Vol. 9, Book 92, Hadith 450). Retrieved July 30, 2020, from https://sunnah.com/bukhari/96/79.

[CIT0046] Mustafa, Y. (2014). Islam and the four principles of medical ethics. *Journal of Medical Ethics*, *40*(7), 479–483. 10.1136/medethics-2012-101309 23975951

[CIT0047] Nusseibeh, S. (2017). *The story of reason in Islam*. Stanford University Press.

[CIT0048] O’Connor, A. E., Winch, S., Lukin, W., & Parker, M. (2011). Emergency medicine and futile care: Taking the road less travelled. *Emergency Medicine Australasia*, *23*(5), 640–643. 10.1111/j.1742-6723.2011.01435.x 21995480

[CIT0049] Rachels, J. (2001). Killing and letting die. In L. C. Becker, & C. B. Becker (Eds.), *Encyclopaedia of ethics* (Vol. 2. 2nd ed., pp. 947–950). Routledge.

[CIT0050] Sachedina, A. (2006). The search for Islamic bioethics principles. In R. E. Ashcroft (Ed.), *Principles of health care ethics* (pp. 117–125). John Wiley & Sons.

[CIT0051] Sachedina, A. (2009). *Islamic biomedical ethics*. Oxford University Press.

[CIT0052] Sen, A. (1983). Evaluator relativity and consequential evaluation. *Philosophy & Public Affairs*, *12*(2), 113–132.

[CIT0053] Serour, G. I. (1994). Islam and the four principles. In R. Gillon, & A. Lloyd (Eds.), *Principles of health care ethics* (pp. 75–91). John Wiley & Sons.

[CIT0054] Swetz, K. M., Burkle, C. M., Berge, K. H., & Lanier, W. L. (2014). Ten common questions (and their answers) on medical futility. *Mayo Clinic Proceedings*, *89*(7), 943–959. 10.1016/j.mayocp.2014.02.005 24726213

[CIT0055] Taymiyyah, I. (n.d.). *Majmūʿ Fatāwā al-Kubra (Vol. 4)*. Matbaʿat Kurdistan.

[CIT0056] White, B., Willmott, L., Close, E., Shepherd, N., Gallois, C., Parker, M. H., Winch, S., Graves, N., & Callaway, L. K. (2016). What does “futility” mean? An empirical study of doctors’ perceptions. *Medical Journal of Australia*, *204*(8), 318. 10.5694/mja15.01103 27125807

[CIT0057] Winkler, E. (1995). Reflections on the state of current debate over physician-assisted suicide and euthanasia. *Bioethics*, *9*(3), 313–326. 10.1111/j.1467-8519.1995.tb00367.x 11653048

[CIT0058] World Health Organization. (n.d.). *Palliative care*. Retrieved July 30, 2020, from https://www.who.int/cancer/palliative/definition/en/.

